# The moderating effect of psychological trust on knowledge spillovers and firms’ open innovation

**DOI:** 10.3389/fpsyg.2022.1071625

**Published:** 2022-12-13

**Authors:** Rui Huang, Jie Jin, Tianxin Sunguo, Yongsong Liu

**Affiliations:** ^1^International Business School, Yunnan University of Finance and Economics, Kunming, China; ^2^Division of Science & Technology Administration, Yunnan University of Finance and Economics, Kunming, China; ^3^Business School, Yunnan University of Finance and Economics, Kunming, China; ^4^School of International Languages and Cultures, Yunnan University of Finance and Economics, Kunming, China

**Keywords:** knowledge spillover, firms’ open innovation, psychological trust, explicit knowledge spillover, tacit knowledge spillover

## Abstract

Psychological trust is an important link in building interpersonal relationships and has a significant impact on the attitude and behavior of knowledge subjects. Based on the characteristics of knowledge attributes, this paper analyzed the data of 180 high-tech firms in China from 2014 to 2020 to deeply explore the effects of explicit knowledge spillover and tacit knowledge spillover on firms’ open innovation, and the moderating effect of psychological trust on the relationship between the two. It is found that: first, explicit knowledge spillover and tacit knowledge spillover have an inverted U-shaped relationship with firms’ open innovation, i.e., the effect of open innovation increases and then decreases as the degree of knowledge spillover increases; second, psychological trust positively moderates the non-linear relationship between knowledge spillover and firms’ open innovation. This paper provides a rational explanation of firms’ management behavior from a psychological perspective, and enriches and expands the research related to knowledge spillover, firms’ open innovation and psychological trust. It is suggested that firms should pay more attention to inter-organizational trust relationships and pay attention to the psychological growth and development of knowledge employees to improve open innovation in firms.

## 1 Introduction

With the explosion of information and accelerated global innovation in the knowledge economy, it is challenging for firms to hold the complete resources and technologies required for innovation (Flipse et al., 2013). What’s more, firms with core knowledge in a particular domain of expertise are also at risk of being disrupted or overturned. It is urgent for firms to change their development paradigm as the “closed innovation model” that completely relies on the firm’s internal resources has failed to follow on the heels of an increasingly complex market and technological environment. Chesbrough and Crowther (2006) proposed that as an innovation model, open innovation can help firms to cross the innovation boundary and to achieve innovation goals while strengthening the flow of knowledge resources among innovation subjects. The research shows that as a central innovation strategy and innovation model for firms, open innovation plays a key role in innovation activities (Yang et al., 2021). Especially in the context of economic globalization, open innovation and activities are growing. As the world’s largest emerging economy, Chinese firms are going global at an accelerating pace, and they need to be more open to the rapid updating of technological knowledge and strive to capture complementary resources in open innovation in order to gain sustainable competitive advantages and further enhance the international competitiveness of Chinese firms. Obviously, as a new innovation model, open innovation is receiving more and more attention at the theoretical and practical levels, but how to improve the effectiveness of open innovation in firms has been analyzed by academics.

Previous studies have shown that firms can acquire knowledge resources spilled outward from other knowledge subjects in direct or indirect communication and interaction (Yi et al., 2021). As a critical element of firm knowledge management, the relationship between knowledge spillover and innovation has received widespread attention from academics (Bloom et al., 2013). Aghion et al. (2014) recommend that knowledge spillovers from advanced firms provide more possibilities for others to “learn by doing,” thus increasing the probability of successful innovation. In addition, firms can proactively seek potential collaborators by selectively sharing part of their knowledge (Alexy et al., 2013) to avoid the risk of core technology leakage, Hence, an effective source of innovation is provided by knowledge spillover for firms’ open innovation. Conversely, instead of solely considering how the recipient firm can benefit from the knowledge spillover process (Zahra and George, 2002), recent research shows that while the recipient firm utilizes the spilled knowledge, firms on the spillover producer also have the potential to benefit (Yang and Steensma, 2014). Simultaneously, knowledge external interaction exploration in the knowledge spillover process provides valuable learning opportunities for spillover firms to enhance innovation ability (Xin Ding et al., 2010). Accordingly, systematic and comprehensive consideration of the impacts on both sides of knowledge spillover in the spillover process is also an essential premise for the exploration of the relationship between knowledge spillover and firms’ open innovation.

Moreover, the effectiveness of knowledge spillover may be affected by context factors, such as the organizational atmosphere (Kim and Park, 2020). Filatotchev et al. (2011) believed that it is difficult to separate knowledge from those who possess it since the majority parts of the knowledge within firms are complicated and tacit. The skills of the knowledge subject play a key role in achieving the knowledge spillover effect (Ramadani et al., 2017). Lin et al. (2012) proposed that in a such social phenomenon, interpersonal and social relations are essential factors to promote the emergence of knowledge spillover. The psychological trust of employees can promote not only social collaboration but also close and frequent interaction between individuals. As a psychological state, psychological trust is an essential bound to establish and cultivate interpersonal and social relations (Dirks and Ferrin, 2001). By establishing proactive psychological contracts with employees, firms change employees’ attitudes and behaviors, including their motivation, ability and willingness to engage in knowledge exchange (Malik and Nilakant, 2016). Consequently, psychological trust is a psychological mechanism for deepening knowledge exchanges (Colquitt et al., 2012), and its importance in the spillover of knowledge cannot be ignored. Therefore, this paper explores the role of psychological trust between knowledge spillover and firms’ open innovation.

In summary, although the research related to the impact of knowledge spillovers on innovation has been around for a long time, the existing research rarely mentioned the relationship between knowledge spillover and a specific innovation model, such as open innovation; furthermore, prior research has trapped into analyzing the effects of knowledge spillover from the perspective of the recipient, while the influence on the spillover producer has been ignored. However, research has shown that in the process of knowledge spillover, the spillover producer also has an indirect impact on the innovation effect. In addition, regardless psychological change is an important factor influencing individual behavior, the existing literature provides poor evidence on whether and how psychological factors influence knowledge spillover activities and open innovation activities within firms. Based on the above analysis, this paper integrates knowledge management theory and innovation theory, focusing on the changes influenced during the process of knowledge spillover of both recipient and spillover producers, this paper explores the potential relationship between knowledge spillover and open innovation in firms from the perspective which regards knowledge spillover as an essential knowledge acquisition channel. Moreover, in order to enrich and expand research in related fields from the perspective of employee psychology, psychological trust is introduced as a specific psychological factor in this paper to observe its moderating effect on the relationship between the two mentioned above.

The rest of the paper is organized as follows: The second section explores key foundational theories. Meanwhile, research hypotheses and models are proposed. The third part is the methodology which includes the construction of the regression model, variable design, variable measurement and sample selection. The fourth section summarizes the findings of the empirical analysis, including the robustness test, statistical analysis and analysis results. Finally, the discussion and conclusion consist of research conclusions, theoretical contributions, implications, limitations and prospective direction for future research.

## 2 Literature review

### 2.1 Open innovation

Chesbrough first introduced the concept of open innovation and contended that open innovation is a new paradigm of innovation management in which firms maximize the use of resources inside and outside the organization to innovate and earn profit. Guo et al. (2019) pointed out that in a business environment, innovation resources and experience will be gained through accelerated openness once a firm is under the pressure of a competitor. Regarded as interaction, integration and synergy between elements of innovation, open innovation realizes the free flow of crucial resources to cross the organizational boundary (Ritala et al., 2017). Although scholars have reached a general consensus on the importance of open innovation while a unified standard for related concepts is still missing. Alam et al. (2022) considered that the mechanism of the role of open innovation cannot be fully explained from the single view of firms that a study in which the management theory and the resource dependency theory should be conjoined. Based on the above research, this paper analyzes the concept of open innovation from diverse perspectives by summarizing the existing literature and concludes with the following aspects: (1) From the perspective of innovation resource, resource theory regards open innovation as an asset. Vanhaverbeke et al. (2008) deem that the acquisition of productive resources such as knowledge, customers or suppliers, and infrastructure services is the main purpose of open innovation; (2) From the perspective of knowledge, Lichtenthaler (2009) mentioned that open innovation is a process of knowledge search and enrichment, in which internal and external knowledge development, integration and utilization are systematically carried out through innovation activities; (3) From the perspective of resource dependence theory, the inability of firms to fully integrate the entire resources needed for innovation by internal integration leads to the inevitable demands of production resources provided by other firms (Chesbrough, 2003).

In addition, the influencing factors of firms’ open innovation are also highlighted in this paper. (1) Firm’ capability. Keupp and Gassmann (2009) thought that a series of innovation obstacles, such as rising R&D costs within firms and the shortening trend of the product life cycle, make certain firms more inclined to carry out open innovation than others; (2) Organizational Environment. In the process of innovation, Chesbrough and Crowther (2006) considered that external resources and knowledge contribute to innovation performance even in firms with strong R&D and innovation capabilities; (3) Senior management characteristics. According to Salter et al. (2014), the thinking ability and innovative ideas of R&D individuals have a nonlinear relationship with the openness of external knowledge sources. Meanwhile, the knowledge-based theory shows that knowledge, as the core element of innovation, is the basis of open innovation (Hilbert and López, 2011). Ye (2022) deemed that the improvement of innovation capabilities depends not only on the firm’s R&D investment but also on the diffusion or spillovers of external knowledge and R&D capital. Knowledge spillovers with no compensation or compensation less than the actual value of knowledge can acquire valuable core technologies from other firms (Cassiman and Veugelers, 2002). Through knowledge spillovers, the knowledge base and resources of other firms are used to increase firms’ own R&D investment and to achieve more innovative output with less R&D cost (Dai et al., 2022). As the unique modality of knowledge acquisition makes knowledge spillover an essential factor affecting open innovation, this paper analyzes how to optimize the use of knowledge spillover to promote firms’ open innovation from the perspective of knowledge spillover.

### 2.2 Knowledge spillover

Since the concept of knowledge spillover is introduced in the analysis of economic problems by Arrow in 1962, knowledge spillover has become an important research direction in economics and management. However, concepts related to knowledge spillover such as knowledge diffusion, knowledge transfer and knowledge flow are confused by scholars in various fields, in fact, these concepts are quite different. First, knowledge spillover and knowledge transfer belong to the same category of knowledge flow. Fallah and Ibrahim (2004) put forward that knowledge communication may occur in every interaction between knowledge subjects. “Knowledge transfer” is considered to be the knowledge exchange that occurs consciously among people or organizations while any unconscious knowledge transmission beyond such communication belongs to “knowledge spillover.” Whereas, contradictory to previous studies that regard knowledge spillover as an unconscious knowledge dissemination process, Alexy et al. (2013) found that to augment the possibility of acquiring valuable knowledge in the future, firms can consciously select some internally developed knowledge to provide free use to external participants through carriers such as technical drawings, meeting minutes and contracts. pointing out that defining knowledge spillover in terms of “conscious and unconscious” is not accurate. Furthermore, Keller (2010) emphasized that as a method of knowledge diffusion, knowledge spillover mainly refers to the part of knowledge that diffused through externalities. The externality feature of knowledge spillover suggests that knowledge recipients can develop and create new knowledge by combining the acquired knowledge with their knowledge without compensating the knowledge creator or below the compensation of knowledge creation cost (Zhu and Xu, 2019). In a word, this paper states that knowledge spillover refers to knowledge being acquired by subjects other than the knowledge creator in the form of no compensation or compensation less than the value of knowledge created.

Moreover, Angeles Montoro-Sánchez et al. (2011) contended that distinct types of knowledge spillovers lead to differences in the quantity and quality of external resources acquired by firms, which may eventually affect the effect of firms’ innovation. According to the source of knowledge spillover, knowledge spillover can be classified as domestic knowledge spillover and foreign knowledge spillover (Chen et al., 2012). According to the direction of spillover, it is categorized as outward and inward knowledge spillover by Cassiman and Veugelers (2002). Gaur et al. (2019) asserted that knowledge flow depends on the attributes and context of knowledge being transferred. The characteristics of tacit and explicit are the most common factors to affect knowledge flows (Dhanaraj et al., 2004). Jensen (2007) classified knowledge into explicit and tacit knowledge based on the degree of modifiability. As formalized knowledge, explicit knowledge is easy to encode, retrieve and transfer (Hansen, 2002). Inversely, as an informal form of knowledge-based primarily on personal experience and skills, tacit knowledge is difficult to compile and communicate (Bibi and Ali, 2017; Bogers et al., 2018). The huge difference between explicit knowledge and tacit knowledge leads to distinct spillover effects. Consequently, for the purpose of launching an advanced exploration related to the distinct effects of various types of knowledge spillovers on firms’ open innovation, this paper divides knowledge spillover into two dimensions: tacit knowledge spillover and explicit knowledge spillover. In specific, explicit knowledge spillover refers to new technologies or products carried by language, text or graphics that are acquired by other subjects other than knowledge creators for free or at a small cost. Tacit knowledge spillover refers to all kinds of information, experience and skills based on individuals acquired by other subjects other than knowledge creators for free or at a small cost.

### 2.3 Knowledge spillovers and firms’ open innovation

Knowledge spillover is considered an inevitable knowledge transfer phenomenon in open innovation (Cassiman and Veugelers, 2002). Specifically, Knowledge spillover not only provides the raw materials needed for open innovation but also strengthens the communication between the firm and external knowledge, as well as the increment of value extension on spilled knowledge (Yang et al., 2010). However, open innovation is affected differently due to distinct attributes of knowledge. Seidler de Alwis and Hartmann (2008) believed that compared with explicit knowledge, tacit knowledge capable of adapting to the rapidly updating trend of innovative technologies in the knowledge economy plays a more significant role in knowledge spillovers and innovation. Based on the statement above, this paper explores the role played by tacit knowledge spillover and explicit knowledge spillover in open innovation.

#### 2.3.1 Explicit knowledge spillover and firms’ open innovation

The majority of explicit knowledge spillovers come from leasing new equipment or purchasing new products from competitors. Although those firms that adopt knowledge spillovers to create more advanced technologies by using new technologies to process and improve old technologies can avoid the risks of R&D and the entire consequences of R&D failures (Yi et al., 2021). However, new technologies that can be easily obtained induce the appearance of firms’ dependence and the abandonment of risky innovation and R&D. Thus, this paper assumes that explicit knowledge spillovers have a nonlinear relationship with firms’ open innovation.

First, the knowledge spillover producers can take advantage of potentially beneficial learning opportunities from the spillover process (Yang et al., 2010). With the increase of knowledge spillover, it is beneficial for the producers to observe how the recipients combine their knowledge with other new knowledge, stimulate new thinking and new ideas in the spillover firms, and then improve their innovation behaviors and achieve more innovative knowledge reorganization (Dosi, 1988; Yang and Steensma, 2014; Duan et al., 2021), providing an open exchange between innovative knowledge provides a good foundation. Second, knowledge spillover will also produce a demonstration effect (Ramadani et al., 2017), in which advanced products and services will create a sense of crisis and competition awareness for the recipients. As the degree of such spillover increases, the pressure felt by firms will also increase, which will boost their innovation enthusiasm and provide a good basis for the exchange between different knowledge subjects of open innovation; Third, firms are able to strategically promote technology replication, actively shape the cooperative behavior of the others innovation ecosystem and actively guide other players to adopt follow-through strategies thus ultimately influence the industry standard when new equipment and new products are learned and utilized by others (Alexy et al., 2013). In this case, open innovation cooperation will be promoted due to the narrowing of the technological distance between the two sides of knowledge spillovers and the assimilation of development goals. Therefore, collaboration and communication between the source of the spillover producers and the recipients expand as explicit knowledge spillover rises. Open innovation will consequently become more efficient.

However, the negative effect on open innovation appears as the degree of knowledge spillover continues to increase, as the explicit knowledge spillovers will be influenced by other factors, and the difficulty of cooperation and communication between firms’ augments. First, the explicit character of knowledge leads to knowledge transfer at a negligible cost, resulting in “free-rider” behavior (Nieto and Quevedo, 2005). When knowledge recipients realize that they can survive by absorbing external knowledge, they will relax the R&D activities of independent innovation, and excessive knowledge spillover will have a squeezing effect on independent innovation. Firms will ultimately lose their competitive advantages, fail to provide valuable information to other cooperative firms, and eventually withdraw from the market, which is not conducive to the exchange and collaboration of open innovation. The R&D activities of independent innovation will be relaxed when the knowledge recipients realize that it is possible to survive by absorbing external knowledge. This means that excessive knowledge spillover causes a squeezing effect on independent innovation. The knowledge spillover producer will not only lose their own competitive advantages but also be eliminated from the market over time as valuable information to other cooperative firms cannot be provided, which is not conducive to the exchange and cooperation of open innovation. What’s more, Harabi (1995) emphasized that it is impossible for firms to acquire external knowledge for free. Only firms that have accumulated a large amount of relevant knowledge internally can be able to absorb and use such proprietary technology. The more explicit knowledge is accepted, the more the firm needs to spend on identifying, assimilating, and integrating external knowledge, which is likely to result in costs over benefits. Based on the consideration of maximizing benefits, instead of new external information acceptance or open innovation collaboration, firms tend to innovate alone. Based on the above analysis, this paper puts forward the following hypotheses:

H1: There is an inverted U-shaped relationship between explicit knowledge spillover and firms’ open innovation. In other words, explicit knowledge spillovers promote open innovation until the inflection point is reached. Once the inflection point is reached, firms’ open innovation starts to decline.

#### 2.3.2 Tacit knowledge spillover and firms’ open innovation

The characteristics of mute and complexity make it more difficult for tacit knowledge to be codified or communicated. Correspondingly, the task of obtaining valuable tacit knowledge becomes challenging (Umar et al., 2021). According to Seidler de Alwis and Hartmann (2008), tacit knowledge transfer is primarily based on individual interactions and experiences. It is possible to transfer tacit knowledge more successfully through personal mobility (Song et al., 2003) and practical experience. Hence, this paper believes that the mobility and attitudes of individuals play a significant role in the impact of tacit knowledge spillover on firms’ open innovation.

In the initial stage of knowledge spillover, tacit knowledge spillover positively contributes to firms’ open innovation: first, employees can continuously “learn by doing” in new areas through individual mobility to dig the depth and expand the breadth of knowledge, which will eventually accelerate the value addition of individuals and the creation of new knowledge (Choudhury and Kim, 2019). In this process, the knowledge spillover producer will enhance the exploration of new knowledge in pursuit of higher value. Meanwhile, the knowledge recipients can continuously acquire new knowledge spillover, thus improving the level of innovation in the whole organization. Second, the interaction of knowledge is facilitated by the mobility of skilled professionals at various spatial scales (Almeida and Kogut, 1999). Knowledge complementarity between knowledge spillover producers and recipients can promote collaboration and knowledge recombination among knowledge subjects that may stimulate the emergence of more abundant resources (Nyberg and Wright, 2015) and the possibility of firms’ open innovation; Finally, knowledge subjects have strong achievement motivation that aspires to increase higher levels of achievement in work and enhance their value (Aguinis and Glavas, 2012). Showing experience and technology to others and being learned by others meets a higher level of psychological demand for knowledge subjects such as self-fulfillment, being respected or being granted (Krausert, 2014), thus generating a higher accomplishment while the exchanges and cooperation of innovation will be stimulated.

However, the increase in the degree of tacit knowledge spillover brings a more obvious inhibitory effect on open innovation, once the threshold is exceeded. First, excessive tacit knowledge spillover will have competitive effects on open innovation (Zhang et al., 2022). In this situation, the risk of the core technical knowledge of the knowledge spillover producer being leaked increases, and competitors can quickly achieve technological catch-up and product substitution through excessive knowledge transfer and opportunism (Brockman et al., 2018; Ye, 2022), depriving the knowledge producer of their initial competitive advantages and causing the decrescence in employees’ enthusiasm as spillovers for knowledge flow. Second, the technology evolution theory shows that excessive emphasis on prior knowledge would result in technological similarity and incremental evolution, thus the potential knowledge locked-in or path dependence occurs (Burmaoglu et al., 2019). Schilling and Green (2011) contended that valuable innovation often comes from diverse knowledge systems. It is possible that over-absorbing tacit knowledge spillovers from other firms lead to similar innovation paths between firms, arousing the increasement of knowledge substitutability and the reduction of firms’ likelihood of seeking external collaboration. Consequently, firms’ open innovation will be inhibited. Based on the above analysis, this paper puts forward the following hypotheses:

H2: There is an inverted U-shaped relationship between tacit knowledge spillover and firms’ open innovation. In other words, with an increase in tacit knowledge spillover, firms’ open innovation is on the rise; when tacit knowledge spillover exceeds the inflection point level, firms’ open innovation begins to decline.

### 2.4 The moderating effect of psychological trust on knowledge spillover and firms’ open innovation

Scholars have not yet reached a consensus on the definition of trust, due to the characteristics of abstraction and complexity (Rutten et al., 2016). Based on psychology (Lau et al., 2007), individuals generate trust in their psychological consciousness, which is reflected in their positive expectations of other others’ behavior. Jones et al. (2010) suggested that goodwill and competence are two essential components of trust, in which goodwill is typically present in interpersonal relationships and competence is an evaluation of other firms’ skills. The existing literature generally regards trust as a subjective belief that plays a crucial role in facilitating and stabilizing relationships between various subjects. Accordingly, this paper defines psychological trust as a state of psychology, that is, a subject’s optimistic prediction of others’ behavior as well as a subject’s view of the fairness and security of the organizational environment. According to Bottazzi et al. (2016), it is challenging for firms to constrain opportunistic behaviors during open innovation through formal means. Firms engaged in open innovation must simultaneously rely on other means to ease the conflict between various types of knowledge. Thus, during the process of cooperation, psychological trust helps to union different subjects together, to remove informational obstacles and to decrease opportunism. In addition, psychological trust contributes to the establishment of open knowledge environment where a variety of required resources for knowledge subjects are provided (Von Krogh et al., 2012). Inter-firm cooperation based on trust is beneficial to the establishment of an organizational environment for mutual understanding and communication. Such cooperation can motivate organizational members to share their explicit and tacit knowledge (Nonaka et al., 2016; Millar et al., 2017). In this paper, we analyzed the changes in the relationship between knowledge spillover and open innovation in firms under different psychological trust levels.

#### 2.4.1 The moderating effect of psychological trust on explicit knowledge spillover and firms’ open innovation

Explicit knowledge is generally contained in an organization’s accessible artifacts and structural elements. In this regard, companies can obtain valuable explicit knowledge spillovers through convenient measures such as purchasing patents or participating in trade fairs (Lee et al., 2021; Bernal et al., 2022). However, the potential impact of knowledge gaps on spillovers means that larger knowledge gaps come with higher costs and more uncertainty (Bernal et al., 2022). Therefore, the negative knowledge spillover effect happens when a lack of a mutual knowledge foundation occurs, making it difficult for knowledge recipients to absorb and understand the spillover producers’ products, equipment, etc. (Perri et al., 2013). Yuan et al. (2016) concluded that good psychological trust promotes knowledge transfer and sharing. Under the guarantee of the trusted relationship between the two sides of knowledge spillover, the knowledge spillover producers are willing to offer resources and tools required for innovation to the recipients. Under the such relationship, such relationship can shorten the technological distance between firms, accelerate the absorption of valuable innovative products by the recipients, improve the recognition of innovation strategies of both sides of knowledge spillover, and promote the development of firms toward a similar technological path, thus the generation of open innovation will be accelerated. Second, psychological trust helps alleviate the problem of knowledge information asymmetry between firms (Ho et al., 2018). Considerable information asymmetry in innovation activities leads to the possibility that recipients cannot trust the source of knowledge spillovers. The specific performance is that even if new explicit knowledge resources (such as machinery and equipment, innovative products, etc.) that are easy to absorb and understand are obtained, firms still need to spend time and cost on ongoing adaption and adjustment. On the contrary, under the condition of mutual trust, firms can shorten the learning cycle through repeated interactions, thus reducing the coordination costs between partners (Gulati and Singh, 1998). In this way, the process of innovation commercialization is shortened and the formation of open innovation outcomes is accelerated. Based on the above analysis, this paper puts forward the following hypotheses:

H3: Psychological trust strengthens the relationship between explicit knowledge spillover and firms’ open innovation; in other words, psychological trust makes the inverted U-shaped relationship between explicit knowledge spillover and firms’ open innovation steeper.

#### 2.4.2 The moderating effect of psychological trust on tacit knowledge spillover and firms’ open innovation

The trusted relationship between organization members is the basic condition for creating, sharing and using tacit knowledge (Seidler de Alwis and Hartmann, 2008). The connection based on mutual trust and understanding can effectively facilitate the exchange of information and enhance the continuous flow and diffusion of knowledge, especially tacit knowledge spillover. First, a high level of psychological trust creates a cooperative atmosphere which facilitates knowledge spillover and increases knowledge producers’ willingness to spill knowledge (Bibi and Ali, 2017; Park and Kim, 2018). The spillover of tacit knowledge stored in people’s minds and experiences depends on the working practices and face-to-face communication of the subject of knowledge (Nonaka, 2008). Face-to-face communication and guidance between individuals are necessary conditions for the effective spillover of individual unique knowledge (Berraies et al., 2020). Moreover, psychological trust inspires employees to engage in collaborative activities, to share and to absorb knowledge from other trusted individual. In this case, perceptions based on psychological trust not only promote social collaboration but also encourage close and frequent interactions between individuals, between which employees can feel comfortable with sharing their knowledge as their perception of the risk of opportunistic behavior is reduced (Sankowska, 2013); second, the representational gaps are one of the biggest obstacles in knowledge exchange, particularly for tacit knowledge (Cronin and Weingart, 2007). The representational gaps are the differences in perceptions of an issue among various knowledge subjects. As knowledge subjects cannot fluently master each other’s domains of knowledge, diverse perceptions of issues arising from different knowledge and values among individuals in an open innovation challenge have the potential to undermine collective information processing. Sun et al. (2020) thought that building and maintaining trustworthy relationships in knowledge exchange enables firms to overcome the representational gaps that a mutual trust helps to increase the partners’ understanding and appreciation. The effectiveness of knowledge spillover can be enhanced when individuals trust others and show positive attitudes to understand others (Scuotto et al., 2020). Based on the above analysis, this paper puts forward the following hypotheses:

H4: Psychological trust strengthens the relationship between tacit knowledge spillover and firms’ open innovation; in other words, psychological trust makes the inverted U-shaped relationship between tacit knowledge spillover and firms’ open innovation steeper.

**FIGURE 1:**
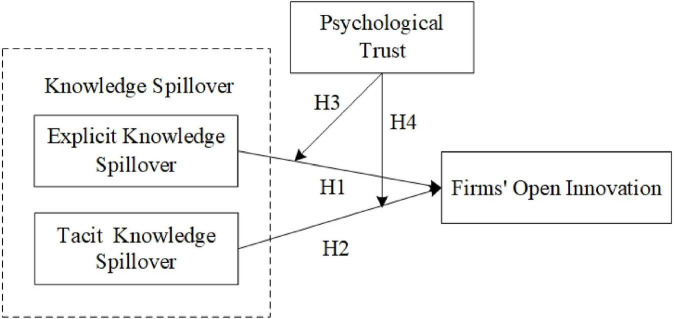
Research framework.

Based on the above analysis, our research model is shown in Figure 1.

## 3 Materials and methods

### 3.1 Data

Duan et al. (2022) thought that manufacturing is an important area of comprehensive national power competition and technological competition among countries in the world. According to the current industrial classification of the Chinese national economy, the high-tech manufacturing industry mainly includes firms in pharmacy, aerospace, transportation equipment manufacturing, computer and other electronic equipment manufacturing. Meanwhile, high-tech manufacturing firms are knowledge-intensive, and their production and operation status, R&D activities, new product development and sales, and patents all account for important ratios (Duan et al., 2021). Based on the above analysis, we collected the financial statements of Chinese A-share listed manufacturing firms in the CSMAR database, the sample firms were screened as follows: (1) Firms with “ST” and “*ST” marks are excluded; (2) Firms in financial industries with apparent differences in accounting standards from other industries are excluded; (3) Firms with missing information are also excluded. Since the primary data for this paper were obtained from the CSMAR database and the 2015 China General Social Survey (CGSS), where the CSMAR database is only updated to 2020 and the CGSS data were collected in 2015, we aimed to measure with the most recent sample data based on the availability and completeness of the data. As a result, the data of 180 firms from 2014 to 2020 were selected as the research sample.

### 3.2 Measures

#### 3.2.1 Dependent variable

Patents are considered the primary manifestation of firms’ open innovation outcomes, referring to Brockman et al. (2018), the quantity of joint patent applications is used to measure a firm’s effectiveness in terms of open innovation. Joint patents are commonly used as a representative of collaborative innovation in the management and organizational literature (Arora et al., 2016), as Joint ownership of intellectual property is an effective strategy for firms to jointly develop technologies. In order to obtain the open innovation performance of the firms, the number of Joint patent applications is used to measure open innovation performance, the standard applicant names of all patents of the sample firms are downloaded from the National Intellectual Property Administration (NIPA), and the number of Joint patents with the number of applications greater than one are filtered out and summed up in this paper.

#### 3.2.2 Independent variable

Most scholars agreed that a firm’s patents are a good indicator to measure knowledge spillovers (Yamin and Otto, 2004). Seidler-de Alwis and Hartmann (2008) assumed that patents are an ideal representation of explicit knowledge in the business environment. Following their approach, this paper measured explicit knowledge spillover by calculating and analyzing the number of patent applications to represent a firm’s knowledge and technological output. The more patent applications a firm has, the more knowledge creation and the more knowledge spillover it generates to the outside. In addition, Block (2012) stated that higher R&D expenditures generate abundant new knowledge, leading to a stronger knowledge spillover effect. Therefore, the level of total R&D expenditures is used as a proxy variable for tacit knowledge spillover in this paper’s empirical study on innovation and knowledge spillover.

#### 3.2.3 Moderating variable

This paper makes use of data from the China General Social Survey (CGSS) 2015 to establish our measure of psychological trust, which is based on Zhang and Ke (2002). In response to the question “In general social interactions/contacts that do not directly involve pecuniary interests, how many strangers do you think you can trust?” The survey respondents were given the options of “Mostly untrustworthy,” “Mostly untrustworthy,” “50-50 between trustworthy and untrustworthy,” “Mostly trustworthy,” “Mostly trustworthy,” and “overwhelmingly credible.” We assign values 1, 2, 3, 4, and 5 to each of these five options to calculate the average value for all residents of each province and city as the trust indicator value for the simple province and city, which is adapted to assign a value to the psychological trust of employees in firms in the province to which they belong. Considering that the trust environment of a region is less likely to change in the short term (Umar et al., 2021), this paper uses the index of 2015 as a proxy for the trust situation in 2014–2020 as well.

#### 3.2.4 Control variable

Based on the existing research results, this paper used the variables of Firm age, Firm size, financial leverage (FL), Firm profitability (ROA) and the number of R&D personnel as control variables that affect the factors of firms’ open innovation and knowledge spillover.

**(1) Firm age.** Young firms have a larger risk of innovation (Chin et al., 2022), and they may be more willing to cooperate. Also, Chesbrough (2003) assumed that older firms have a stronger absorptive capacity, which enables them to better identify, absorb and utilize external knowledge in the knowledge spillover process.

**(2) Firm size.** Prior research has identified firm size as a key driver of innovation collaboration. Smaller firms may have greater resource constraints, therefore, having a greater need for open innovation and a faster reaction toward open decisions (Chesbrough and Crowther, 2006).

**(3) Financial leverage (FL).** A firm’s financial position affects its willingness to engage in open innovation. Firms that are more financially constrained can use relationships from innovation networks to overcome resource constraints and increase productivity (Chesbrough and Crowther, 2006). We consider that the role of firms’ profitability reflects the firm’s financial position.

**(4) Firm profitability (ROA).** The behavior of investing in innovation is influenced by the financial stability and operational performance of the firms (Chin et al., 2021). Firms with high profitability are usually willing to invest more resources in innovation, while firms with weak profitability lack the resources to invest in innovative activities (Liu and Li, 2020). Therefore, profitability may have an impact on open innovation.

**(5) Number of R&D personnel.** Firms that are committed to R&D investment are more possible to have more co-patents (Blundell et al., 1999). Therefore, the importance of controlling a firm’s overall R&D situation drives us to adopt the number of R&D personnel as a control variable.

The definition of each variable in this paper is shown in Table 1.

**TABLE 1:** Variables and measurements.

Variable	Variable name	Variable measurement
Dependent variable	Open innovation (OI)	Number of joint patent applications
Independent variables	Explicit knowledge spillover (EKS)	Total expenditure for R & D
	Tacit knowledge spillover (TKS)	Number of patent applications for firms
Moderating variables	Trust(TR)	Chinese General Social Survey data in 2015
Control variables	RDPerson	Number of R & D personnel
	Financial Leverage (FL)	Total liabilities/Total assets
	Firm profitability (ROA)	Net profit/Total assets
	Firm Age (Age)	The difference between the statistical cut–off year and the establishment date of the firm
	Firm Size (Size)	The natural logarithm of total assets

### 3.3 Model

We analyze the relationships between firms’ open innovation, psychological trust, and knowledge spillover in high-tech manufacturing firms. To test the proposed hypotheses H1-H4, we establish the following regression models:


(1) O⁢Ii,t=α0+α1⁢T⁢K⁢Si,t+α2⁢T⁢K⁢S2i,t   +Σ⁢α*c⁢o⁢n⁢t⁢r⁢o⁢l⁢si,t+εi,t

(2) O⁢Ii,t=β0+β1⁢E⁢K⁢Si,t+β2⁢E⁢K⁢S2i,t   +Σ⁢β*c⁢o⁢n⁢t⁢r⁢o⁢l⁢si,t+εi,t

To test hypotheses H3,H4, based on the model (1), (2), we add the interaction terms of psychological trust, tacit knowledge spillover, and explicit knowledge spillover square to establish model (3), (4), respectively, as follows:


(3) O⁢Ii,t=λ0+λ1⁢T⁢K⁢Si,t+λ2⁢T⁢K⁢S2i,t+λ3⁢T⁢K⁢Si,t*T⁢Ri,t+λ4⁢T⁢K⁢S2i,t*T⁢Ri,t+λ5⁢T⁢Ri,t+Σ⁢β*c⁢o⁢n⁢t⁢r⁢o⁢l⁢si,t+εi,t

(4) O⁢Ii,t=γ0+γ1⁢E⁢K⁢Si,t+γ2⁢E⁢K⁢S2i,t+γ3⁢E⁢K⁢Si,t*T⁢Ri,t+γ4⁢E⁢K⁢S2i,t*T⁢Ri,t+γ5⁢T⁢Ri,t+Σ⁢β*c⁢o⁢n⁢t⁢r⁢o⁢l⁢si,t+εi,t

## 4 Empirical analysis

### 4.1 Descriptive statistics and correlation analysis

We performed a descriptive statistical analysis of all variables and Table 2 presents the means, standard deviations, minimum and maximum values of the variables. The statistical results show that there is a large gap between the minimum and maximum values of firms’ open innovation. In terms of the two dimensions of knowledge spillover, the mean value of explicit knowledge spillover in firms is higher than that of tacit knowledge spillover, indicating that most firms mainly use explicit knowledge spillover to promote innovation.

**TABLE 2:** Descriptive statistics of variables.

Variable	N	Mean	SD	Min	Max
OI	1246	7.181	22.140	0	296
TKS	1255	18.713	1.373	11.369	22.718
EKS	1221	53.980	114.446	0	1633
Trust	1253	38.195	2.376	32.632	42.198
RP	1256	743.281	1011.194	0	12481
Age	1257	20.486	4.138	9.920	36.330
Size	1253	22.783	1.143	19.910	33.350
Asset	1253	0.459	0.205	0.034	2.290
ROA	1249	0.039	0.076	-0.957	0.340

The matrix of correlation coefficients between the variables is displayed in Table 3. The table demonstrates a significant positive correlation between firms’ open innovation and tacit knowledge spillover (TKS) and explicit knowledge spillover (EKS). Regression analysis must, however, be used to examine the relationship between the independent and dependent variables in further detail. The analysis of the data reveals a significant positive link between psychological trust (TR) and open innovation practices across businesses. The correlation coefficients between the variables were also typically lower than 0.5. However, certain correlation coefficients were higher than 0.5, thus a variance inflation factor test (VIF test) was carried out in this paper to avoid the problem of multicollinearity. Table 4 shows the VIF test results of the variables in which the maximum value of the VIF test result is 2.050 < 5 and the mean value of the VIF test result is 1.59 < 5, so there is no serious co-linearity problem between the variables.

**TABLE 3:** Correlation coefficient matrix of variables.

	OI	TKS	EKS	Trust	RP	Age	Size	Asset	ROA
OI	1								
TKS	0.165***	1							
EKS	0.262***	0.372***	1						
Trust	0.066**	0.018	0.132***	1					
RP	0.057**	0.604***	0.374***	0.064**	1				
Age	0.089***	0.004	–0.029	−0.104***	−0.064**	1			
Size	0.179***	0.609***	0.364***	0.130***	0.508***	0.018	1		
Asset	0.017	0.176***	0.158***	0.003	0.176***	0.066**	0.381***	1.000	
ROA	0.062**	0.165***	0.029	0.098***	–0.001	−0.066**	0.060**	−0.540***	1.000

*p < 0.1, **p < 0.05, and ***p < 0.01.

**TABLE 4:** Variable VIF test.

Variable	VIF	1/VIF
TKS	2.050	0.487
Size	2.010	0.497
Asset	1.860	0.537
RP	1.750	0.571
ROA	1.680	0.595
EKS	1.250	0.797
Trust	1.060	0.945
Age	1.030	0.974
Mean VIF	1.590	

### 4.2 Analysis of regression results

**FIGURE 2:**
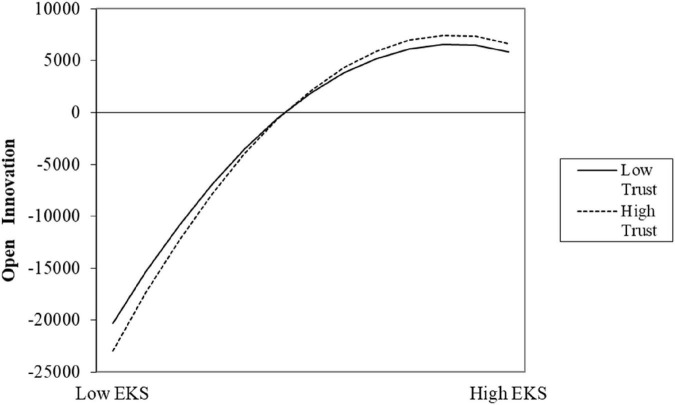
The moderating effect of psychological trust on the relationship between explicit knowledge spillover and open innovation.

**FIGURE 3:**
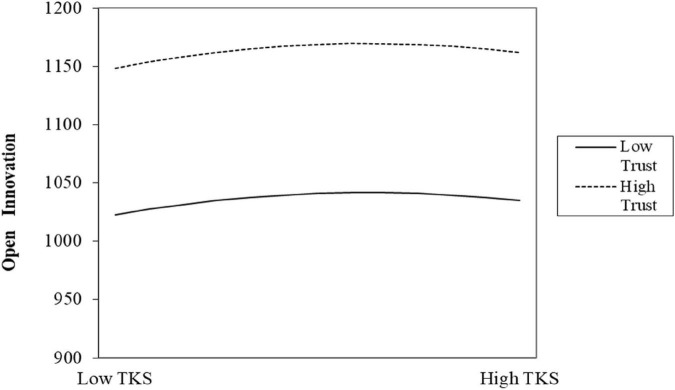
The moderating effect of psychological trust on the relationship between tacit knowledge spillover and open innovation.

Drawing on Haans et al. (2016), this paper proposes three criteria for testing the validity of the inverted U-shaped relationship: first, the primary term coefficient is positive and the quadratic term coefficient is significantly negative; second, the slope at both ends of the definition domain should be significantly positive or negative; and third, the confidence interval at the 95% level of the turning point needs to fall within the definition domain. The test results meet the above requirements, and the details are shown in Figures 2, 3 and Table 5.

**TABLE 5:** Relationships between principal variables.

	(1)	(2)	(3)	(4)
	OI	OI	OI	OI
EKS	0.005***		0.014***	
	(0.001)		(0.001)	
EKS2	−0.000*		−0.000***	
	(0.000)		(0.000)	
TKS		3.648**		10.463***
		(1.722)		(2.308)
TKS2		−0.094**		−0.268***
		(0.046)		(0.060)
Trust			−87.202*	−248.227***
			(46.655)	(66.339)
EKS* Trust			3.059***	
			(0.767)	
EKS2 * Trust			−0.009***	
			(0.001)	
TKS * Trust				4043.868***
				(901.758)
TKS2* Trust				−105.394***
				(23.276)
RP	–3.832	1.609	−0.000***	–0.000
	(5.264)	(8.723)	(0.000)	(0.000)
Age	0.063**	0.067**	0.045***	0.077***
	(0.026)	(0.027)	(0.015)	(0.023)
Size	0.166*	0.483***	0.337***	0.468***
	(0.088)	(0.113)	(0.085)	(0.116)
Asset	–0.400	−0.724*	−1.305***	−1.099**
	(0.400)	(0.409)	(0.367)	(0.459)
ROA	0.444	–0.792	–1.379	–1.846
	(1.082)	(1.063)	(0.883)	(1.200)
Cons	–2.722	−44.753**	−5.671***	−0.217***
	(2.072)	(17.700)	(1.912)	(0.071)
Inflate cons	−0.112*	–0.081	−0.146**	−0.189***
	(0.059)	(0.061)	(0.059)	(0.071)
N	1158	1189	1203	1234
Log pseudolikelihood	–8432.454	–9166.754	–7767.514	–9038.669

Standard errors in parentheses. *p < 0.1, **p < 0.05, ***p < 0.01.

Table 5 shows that Model 1 is a regression of the relationship between the primary term of explicit knowledge spillover, the quadratic term of explicit knowledge spillover, and open innovation of firms. The results show that the coefficient of the primary term of explicit knowledge spillover is: 0.005(*P* < 0.01),and the coefficient of the quadratic term is -0.000 (*P* < 0.1). When the minimum value of explicit knowledge spillover (0), the slope of the curveβ_**1**_ + **2**β_**1**_ = **E***KS*_**m***ax*_ 0.005 > 0; when the maximum value of the explicit knowledge spillover (1633), the slope of the curve β_**1**_ + **2**αβ_**1**_ = **E***KS*_**m***in*_ -0.004 < 0, and the slope of the sample boundary has the opposite sign. And, the inflection point of the curve is 1254.326, which is within the sample range. Open innovation shows an upward and then downward trend with the rise in explicit knowledge spillover of the firm. The inverted U-shaped association between explicit knowledge spillover and open innovation is supported by the data, supporting hypothesis H1. Model 2 is the regression of the relationship between the primary term of tacit knowledge spillover, the quadratic term of tacit knowledge spillover and open innovation. The results show that the coefficient of the primary term of tacit knowledge spillover is: 3.648 (*p* < 0.05) and the coefficient of the quadratic term is -0.094 (*p* < 0.05). When the tacit knowledge spillover takes the minimum value (11.36924), the slope of the curve α_**1**_ + **2**α_**1**_ = **T***KS*_**m***in*_ 1.511 > 0;when the tacit knowledge spillover takes the maximum value (22.71756), the slope of the curve α_**1**_ + **2**α_**1**_ = **T***KS*_**m***ax*_ -0.623 < 0, and the slope of the sample boundary has the opposite sign. The curve’s inflection point, which falls inside the sample range, is 15.87979. There is an inverted U-shaped correlation between tacit knowledge spillover and open innovation, which supports the hypothesis H2 that open innovation shows a tendency of growing and then declining as tacit knowledge spillover of firms grows.

### 4.3 The analysis of moderating effect

According to Haans et al. (2016), the moderating variable can affect the inverted U-shaped relationship in two different ways: first, it can move the turning point to the left or to the right, and second, it can flatten or steepen the curve. The inflection points of the curves are as follows after including the interaction term of psychological trust in models (3) and (4) along with explicit knowledge spillover, tacit knowledge spillover, and their squared terms in the equation.


(5) $$T\,K\,S^{*}=\frac{-\lambda_{1}-\lambda_{3}\times T\,R}{2\,\lambda_{2}+2\,\lambda_{4}\times T\,R},E\,K\,S^{*}=\frac{-\gamma_{1}-\gamma_{3}\,T\,R}{2\,\gamma_{2}+\gamma_{4}\times T\,R}$$

Apparently, when psychological trust shifts, so does the positioning of the inflection point. Calculate the model (5)’s number of partial guidance:


(6) $$\frac{\partial T\,K\,S^{*}}{\partial T\,R}=\frac{\lambda_{1}\,\lambda_{4}-\lambda_{2}\,\lambda_{3}}{2\,{\left(\lambda_{2}+\lambda_{4}\times T\,R\right)}^{2}},\frac{\partial E\,K\,S^{*}}{\partial T\,R}=\frac{\gamma_{1}\,\gamma_{4}-\gamma_{2}\,\gamma_{3}}{2\,{\left(\gamma_{2}+\gamma_{4}\times T\,R\right)}^{2}}$$

We set the first derivative with respect to X to zero. Model (6) shifts the curve’s inflection point when it is not equal to 0, **2**(λ_**2**_ + λ_**4**_ = **T***R*)^**2**^ and **2**(γ_**2**_ + γ_**4**_ = **T***R*)^**2**^ are always greater than 0. Therefore, when λ_**1**_λ_**4**_−λ_**2**_λ_**3**_ 0 and γ_**1**_γ_**4**_−γ_**2**_γ_**3**_ 0, the inflection point shifts to the left; λ_**1**_λ_**4**_−λ_**2**_λ_**3**_ 0 and γ_**1**_γ_**4**_−γ_**2**_γ_**3**_ 0 the inflection point shifts to the right. Hypotheses H3 and H4 demonstrate that psychological trust plays a moderating role in the inverted U-shaped relationship between explicit knowledge spillover and tacit knowledge spillover and open innovation. The higher the level of psychological trust, the stronger the positive impact of explicit and tacit knowledge spillover on open innovation. By performing the same test for model (3) and calculating λ_**1**_λ_**4**_−λ_**2**_λ_**3**_ 0 and γ_**1**_γ_**4**_−γ_**2**_γ_**3**_ 0 therefore the whole curve shifts to the left. The coefficients of the psychological trust moderating term are: -87.202 (*p* < 0.1), -248.227 (*p* < 0.01) respectively, which proves the existence of a moderating effect. Columns (3) and (4) in Table 5 show the model regression results after adding the interaction terms of explicit knowledge spillover, tacit knowledge spillover and its quadratic term with psychological trust, respectively. The interaction coefficient between psychological trust and EKS is 3.059 (p < 0.01), while the coefficient of the quadratic term is -0.009 (*p* < 0.01); the coefficient of the interaction term between psychological trust and TKS is 4043.868 (*p* < 0.01) and the quadratic term coefficient was -105.394 (*P* < 0.01). Therefore, the inverted U-shaped relationship between explicit knowledge spillover, tacit knowledge spillover, and open innovation remains valid after including the interaction terms. We plotted the moderating effects of psychological trust based on the results in Table 5 (Figures 2, 3).

Before taking into consideration the ease of psychological trust, Figure 2 illustrates changes in the relationship curve between explicit knowledge spillover and open innovation: (1) Before the inflection point, the curve for psychological trust with a lower level is relatively flat, and the curve for psychological trust with a higher level is steep, suggesting that firms with high psychological trust improve their open innovation performance more quickly as explicit knowledge spillover increases. (2) The inflection point’s location shifts. The apex of the high psychological trust curve is higher than the inflection point of the low psychological trust curve, demonstrating that explicit information spillover is more beneficial to improving open innovation performance at higher levels of psychological trust. (3) After the inflection point, the open innovation curve of the firms has begun to decline. Although the flatness of the various psychological trust curves is comparable, the high psychological trust curve is always higher than the low psychological trust curve, showing that open innovation performance decreases with increasing explicit knowledge spillover regardless of psychological trust; even with the same explicit knowledge spillover, the high psychological trust firms still outperform low trust firms in terms of open innovation performance. The relationship between explicit knowledge spillover and open innovation cannot be considered to be positively regulated by psychological trust because this regulatory effect is dependent on the amount of explicit knowledge spillover. In the link between the two, psychological trust has a negative impact at the level of low explicit knowledge spillover. When the level of explicit knowledge spillover is high, psychological trust positively moderates the relationship between the two.

Calculating coefficient λ_**1**_λ_**4**_−λ_**2**_λ_**3**_ 0, results in the turning point of the curve moving to the left, as shown in Figure 3. The curve shows that regardless of psychological trust, open innovation performance declines when tacit knowledge increases; even if the same tacit knowledge overflows, high psychological trust firms do better in open innovation than low psychological trust ones. H4 is therefore confirmed.

### 4.4 Robustness test

To test the reliability of the regression model and empirical results, this paper uses two ways to test the robustness of the regression results: (1) changing the regression model, i.e., using Poisson regression; (2) lagging the explanatory variable firms’ open innovation by one period, and the robustness results are shown in Tables 6, 7: Model 1 and Model 2 are regressions of the relationship between explicit knowledge spillover, tacit knowledge spillover quadratic term and open innovation, respectively. The results show that the primary coefficients of the explicit knowledge spillover quadratic term are both -0.000 and significant at the 1% and 10% levels, respectively. Also, primary coefficients of the tacit knowledge spillover quadratic term are -8.793 and -0.101 and significant at the 5% and 10% levels, respectively, and hypothesis H1 and hypothesis H2 are verified. Model 3 adds the interaction term of explicit knowledge spillover and psychological trust on the basis of model 1, and the results show that the coefficients of the interaction term of the secondary term of explicit knowledge spillover and psychological trust are -0.008 and -0.006, and both of them are significant at the 1% level, saying that the inverted U-shaped relationship between explicit knowledge spillover and firms’ open innovation is strengthened with the increase of psychological trust; model 4 adds Model 4 adds the interaction term of tacit knowledge spillover and psychological trust on the basis of model 2, and the results show that the coefficients of the interaction term of tacit knowledge spillover and psychological trust are -15.230 and -99.530, which are significant at the 5% and 1% levels, respectively, indicating that the inverted U-shaped relationship between tacit knowledge spillover and firms’ open innovation is strengthened with the increase of psychological trust. The above findings are generally consistent with the regression results and the signs of each variable are the same as the empirical results in the previous paper, proving that the conclusions are robust.

**TABLE 6:** Robustness test: Change regression method.

	(1)	(2)	(3)	(4)
	OI	OI	OI	OI
EKS	0.010***		0.018***	
	(0.000)		(0.001)	
EKS2	−0.000***		−0.000***	
	(0.000)		(0.000)	
TKS		52.952***		1.142**
		(20.329)		(0.505)
TKS2		−8.793**		−0.028**
		(3.534)		(0.013)
Trust			–88.616	−258.634***
			(71.704)	(77.014)
EKS* Trust			1.891***	
			(0.222)	
EKS2 * Trust			−0.008***	
			(0.001)	
TKS * Trust				606.344**
				(261.059)
TKS2* Trust				−15.230**
				(6.927)
RP	0.000***	0.000***	0.000***	0.000***
	(0.000)	(0.000)	(0.000)	(0.000)
Age	0.035***	0.103***	0.010	0.115***
	(0.008)	(0.009)	(0.008)	(0.008)
Size	0.216***	–0.677	0.171***	–0.015
	(0.051)	(1.393)	(0.051)	(0.056)
Asset	1.042***	−0.182***	0.571***	1.589***
	(0.184)	(0.019)	(0.191)	(0.185)
ROA	1.098***	0.049**	0.571*	1.987***
	(0.308)	(0.020)	(0.313)	(0.299)
Cons	−5.039***	−77.310**	−3.022***	–1.200
	(1.060)	(30.579)	(1.057)	(1.203)
N	1203	1112	1203	1234

Standard errors in parentheses. *p < 0.1, **p < 0.05, ***p < 0.01.

**TABLE 7:** Robustness test: Lag open innovation by one period.

	(1)	(2)	(3)	(4)
	OI	OI	OI	OI
EKS	0.005***		0.012***	
	(0.001)		(0.001)	
EKS2	−0.000*		−0.000***	
	(0.000)		(0.000)	
TKS		3.968*		9.314***
		(2.131)		(2.382)
TKS2		−0.101*		−0.235***
		(0.056)		(0.062)
Trust			–56.353	−256.281***
			(53.575)	(68.052)
EKS* Trust			2.173***	
			(0.763)	
EKS2 * Trust			−0.006***	
			(0.001)	
TKS * Trust				3837.871***
				(948.913)
TKS2* Trust				−99.530***
				(24.751)
RP	–12.202	–2.319	−0.000***	–0.000
	(10.562)	(11.538)	(0.000)	(0.000)
Age	0.052**	0.053**	0.032*	0.058**
	(0.026)	(0.027)	(0.017)	(0.025)
Size	0.114	0.393***	0.317***	0.319***
	(0.084)	(0.118)	(0.077)	(0.121)
Asset	−0.992**	−1.284***	−1.680***	−0.959**
	(0.435)	(0.435)	(0.437)	(0.381)
ROA	–0.191	–1.594	−2.615**	–0.000
	(1.332)	(1.275)	(1.258)	(0.000)
Cons	–1.097	−46.012**	−4.683***	−6.075**
	(2.050)	(21.647)	(1.752)	(2.790)
Inflate cons	−0.119*	–0.097	−0.132**	−0.212***
	(0.063)	(0.065)	(0.063)	(0.073)
N	1023	1055	1029	1060
Log pseudolikelihood	–6784.252	–7389.833	–6212.515	–7138.477

Standard errors in parentheses. *p < 0.1, **p < 0.05, ***p < 0.01.

## 5 Discussion

### 5.1 Conclusion

Collected from Chinese listed manufacturing firms in Shanghai and Shenzhen A-shares from 2014 to 2020 as research samples, this paper systematically expounds on the impact of knowledge spillovers on firms’ open innovation. Dividing knowledge spillover into explicit knowledge spillover and tacit knowledge spillover, psychological trust, an important variable in the field of psychology, is introduced as a moderator variable to analyze the influence mechanism of these two different types of knowledge spillovers on firms’ open innovation. The following findings were obtained:

First, this paper found the “double-edged sword” effect of knowledge spillovers on firms’ open innovation. On the one hand, knowledge spillovers can significantly improve the innovation process by lowering R&D costs and market risks; on the other hand, knowledge spillover could hinder both the recipient firm’s ability to innovate and the spillover firm’s passion for innovation. This paper proposed and verified the hypothesis that an inverted U-shaped relationship exists between explicit knowledge spillovers, tacit knowledge spillovers and open innovation. The empirical results demonstrated that the promotion effect of knowledge spillover on firms’ open innovation only functions at a limited level that excessive knowledge spillover damages the promotion effect of open innovation.

Second, although we conclude that knowledge spillover and firms’ open innovation are nonlinear related, different types of knowledge spillover have distinct mechanisms to influence open innovation. In contrast, tacit knowledge spillover takes individuals with knowledge as carriers to acquire valuable experience and technology, which has a subtle impact on open innovation. Meanwhile, we propose that knowledge spillover producers are not always in a disadvantageous position in the spillover process, and proper knowledge exchange is also beneficial to knowledge creators.

Third, the findings indicated that psychological trust is a crucial moderator in the relationship between information spillover and firms’ open innovation. Employees will face resource loss owing to excessive emotional resource consumption when psychological trust is low (Hobfoll, 2001; Halbesleben et al., 2014). On the contrary, the increment of psychological trust can not only promote the exchange of experience and knowledge among organizational members to a large extent but also improve the willingness of organizational members to share knowledge (Yuan et al., 2016). By strengthening and fostering trust amongst knowledge subjects, firms may encourage more collaborative communication and enhance the effectiveness of open innovation.

### 5.2 Theoretical contributions

First, regarding the influence mechanism of knowledge spillovers, this paper breaks through the discussion of a single level that knowledge spillovers only positively or negatively affect firms’ open innovation. While the inverted U-shaped relationship between explicit knowledge spillover, tacit knowledge spillover and firms’ open innovation is verified, the balance of positive and negative effects of knowledge spillover in different contexts is also explored in this paper. As a further refinement of the relationship between knowledge spillovers and innovation, these findings not only help researchers understand the relationship between knowledge spillovers and firms’ open innovation more comprehensively, but also improve knowledge management theory and innovation theory. Moreover, emphasis on the dynamic role of the knowledge spillover producers in the process of knowledge spillover shows that the knowledge spillover producers, as the initiator of knowledge spillover and the bearer of knowledge spillover, has a decisive role in the scale and channel selection of knowledge spillover.

Second, by integrating knowledge spillover, firms’ open innovation and psychological trust into the same framework for research, this paper provided a new understanding vision for the development of open innovation in specific contexts. From the perspective of psychological trust, which effectively combines management science and psychology, we examined psychological changes in knowledge subjects between knowledge spillover and firms’ open innovation. Encouraging the integration of knowledge across disciplines, the above perspective further verified the logical analysis that psychological trust can increase organizational competitiveness more thoroughly while a reasonable explanation of management behavior is provided. What’s more, a rational explanation of management behavior from the perspective of psychology enriches and expands the related research on psychological trust.

### 5.3 Managerial implications

First, as the main subject of innovation activities, firms need to raise their awareness of open innovation and strive to break their own development boundaries. Firms actively cooperate with universities, research institutions and even competitors to obtain the complementary resources they need. Also, a comprehensive and integrated understanding of the relationship between knowledge spillovers and open innovation is necessary for market innovation activities. At the micro level, spillovers are not entirely harmful to firms on the knowledge spillover producers. Turning passive into active, the observation of innovation activities or other knowledge reorganization behaviors that happens in recipient firms can help the knowledge spillover producers to accumulate relevant innovation experience as well as reduce the uncertainty and risk in the subsequent innovation process. Accordingly, recipient firms must be cautious in their resource selection for digestion, and absorption. The reuse of the new external knowledge should be based on their development situation to optimize the benefits of the firms’ knowledge spillover.

Second, firms should pay more attention to the trust relationship between organizations, including the mutual trust between individuals. Furthermore, in addition to paying attention to the trust construction of the entire organization in all aspects of human resource management, the micro-level such as the individual psychology of employees needs more attention. In particular, knowledge-based employees are strongly motivated to achieve their goals with a strong sense of self-worth. To address the needs of knowledge-based talent, firms need to create a competitive compensation and welfare system by focusing on the growth and development of knowledge-based employees.

Third, at the national macro level, market regulators must establish a favorable institutional environment for firms’ innovation. The state must provide knowledge guarantee and institutional support for the transition from Made in China to Create in China, relying on policies and systems such as intellectual property protection to effectively maintain the profits of innovative firms, to limit the “speculative” behavior in innovation activities, and to motivate firms’ innovation activities as well as the whole society.

### 5.4 Limitations and suggestions for future research

First, while this research mainly analyzes the impact of employees’ psychological trust from a static perspective, recent studies indicate that psychological trust shows some fluctuation in the temporal dimension (Qin et al., 2018; Ju et al., 2019). Therefore, future research might investigate the effects of psychological trust in a dynamic temporal context. Second, focused on the positives of psychological trust, potential negatives are neglected. It is possible that psychological trust will lead to emotional tiredness or even destruction. To develop a more comprehensive and dynamic understanding of the relationship between knowledge and innovation, it is meaningful to investigate the detrimental impacts of psychological trust in terms of its function between the two in the future. Third, based on the availability of data, the data sources in this paper cannot be updated to the most recent year, and other measurement methods such as questionnaires or replacement of other databases can be used in the future to make the findings of this paper more comprehensive.

## Data availability statement

The original contributions presented in this study are included in the article/supplementary material, further inquiries can be directed to the corresponding authors.

## Author contributions

RH contributed the central idea, analyzed most of the data, and wrote the initial draft of the manuscript. JJ, TS, and YL contributed to refining the ideas, carrying out additional analyses, and finalizing this manuscript. All authors discussed the results and revised the manuscript.
